# Real-time drilling mud gas monitoring for qualitative evaluation of hydrocarbon gas composition during deep sea drilling in the Nankai Trough Kumano Basin

**DOI:** 10.1186/s12932-014-0015-8

**Published:** 2014-12-16

**Authors:** Sebastian B Hammerschmidt, Thomas Wiersberg, Verena B Heuer, Jenny Wendt, Jörg Erzinger, Achim Kopf

**Affiliations:** MARUM, University of Bremen, Leobener Str., Bremen, 28359 Germany; GFZ German Research Centre for Geosciences, Telegrafenberg, 14473 Germany

**Keywords:** Nankai Trough, Riser drilling, Drilling mud gas monitoring, Mud logging, Kumano basin, IODP, NanTroSEIZE, Accretionary prism, Hydrocarbon gas

## Abstract

**Background:**

Integrated Ocean Drilling Program Expedition 338 was the second scientific expedition with *D/V Chikyu* during which riser drilling was conducted as part of the Nankai Trough Seismogenic Zone Experiment. Riser drilling enabled sampling and real-time monitoring of drilling mud gas with an onboard scientific drilling mud gas monitoring system (“SciGas”). A second, independent system was provided by Geoservices, a commercial mud logging service. Both systems allowed the determination of (non-) hydrocarbon gas, while the SciGas system also monitored the methane carbon isotope ratio (δ^13^C_CH4_). The hydrocarbon gas composition was predominated by methane (> 1%), while ethane and propane were up to two orders of magnitude lower. δ^13^C_CH4_ values suggested an onset of thermogenic gas not earlier than 1600 meter below seafloor. This study aims on evaluating the onboard data and subsequent geological interpretations by conducting shorebased analyses of drilling mud gas samples.

**Results:**

During shipboard monitoring of drilling mud gas the SciGas and Geoservices systems recorded up to 8.64% and 16.4% methane, respectively. Ethane and propane concentrations reached up to 0.03 and 0.013%, respectively, in the SciGas system, but 0.09% and 0.23% in the Geoservices data. Shorebased analyses of discrete samples by gas chromatography showed a gas composition with ~0.01 to 1.04% methane, 2 – 18 ppmv ethane, and 2 – 4 ppmv propane. Quadruple mass spectrometry yielded similar results for methane (0.04 to 4.98%). With δD values between -171‰ and -164‰, the stable hydrogen isotopic composition of methane showed little downhole variability.

**Conclusions:**

Although the two independent mud gas monitoring systems and shorebased analysis of discrete gas sample yielded different absolute concentrations they all agree well with respect to downhole variations of hydrocarbon gases. The data point to predominantly biogenic methane sources but suggest some contribution from thermogenic sources at depth, probably due to mixing. In situ thermogenic gas production at depths shallower 2000 mbsf is unlikely based on in situ temperature estimations between 81°C and 85°C and a cumulative time-temperature index of 0.23. In conclusion, the onboard SciGas data acquisition helps to provide a preliminary, qualitative evaluation of the gas composition, the in situ temperature and the possibility of gas migration.

**Electronic supplementary material:**

The online version of this article (doi:10.1186/s12932-014-0015-8) contains supplementary material, which is available to authorized users.

## Background

### Introduction

Beginning in the 1930s, mud logging became a standard technique on drill rigs worldwide [1]. Besides improving safety during riser drilling, mud logging focuses on formation and reservoir evaluation in real-time [1]. These objectives are accomplished by characterizing the cuttings (i.e. small pieces of the formation) and by analyses of the drilling mud gas composition [1]. Cuttings and drilling mud gas circulate upwards with the drilling mud, where the cuttings are collected at the shale shaker and investigated under the microscope [1]. The gas component is removed from the drilling mud by a degasser, and is then forwarded to the mud gas monitoring unit, where the gas composition is analyzed [1].

Causes for varying gas concentrations in the drilling mud gas data are difficult to assess, because drilling mud gas is a function of the *in situ* gas composition, physical and chemical properties of the formation and the drilling mud, and the drilling operation. Gas sources include liberated gas (i.e. gas that is released when the drill bit crushes the rock), produced gas (i.e. gas inflow caused by borehole pressure lower than hydrostatic), atmospheric gas (O_2_, N_2_, Ar), and recycled gas (i.e. gas not liberated at the surface when the mud is collected in the mud pits) [1,2]. The amount of liberated gas strongly depends on the porosity and permeability of the penetrated formation, but also on parameters like rate-of-penetration, mud weight, mud flow rate, bit and borehole diameter, and degasser efficiency [3].

During the last decades, the scientific value of drilling mud gas monitoring was repeatedly highlighted [1,2,4-11], and found also its way into the Integrated Ocean Drilling Program (IODP) Nankai Trough Seismogenic Zone Experiment (NanTroSEIZE). During riser drilling with *D/V Chikyu*, drilling mud gas monitoring was first conducted with third-party tools on IODP Expedition 319 [12]. The third-party installation was later replaced by an onboard scientific drilling mud gas monitoring system (hereafter termed “SciGas system”), which was tested successfully during IODP Exp. 337 for the first time [13]. The SciGas system allows determination of hydrocarbons (methane, ethane, propane, *i*- and *n*-butane, propane), stable carbon isotopic composition of methane (δ^13^C_CH4_), and non-hydrocarbons (e.g., amongst others, O_2_, N_2_, Ar, H_2_, Xe, He) gases. Tests confirmed that the SciGas system yields accurate δ^13^C_CH4_ values (Heuer et al., unpublished data).

**Figure 1:**
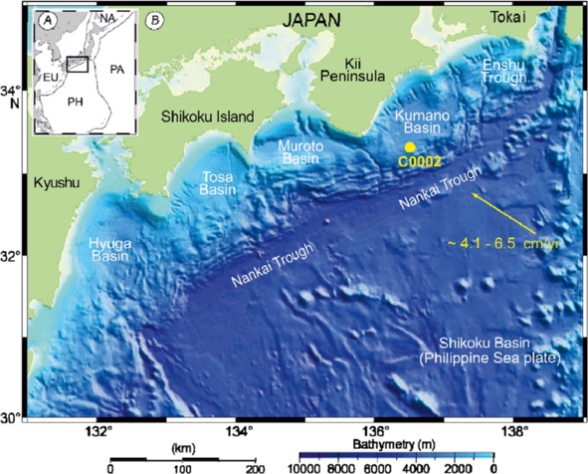
**Overview of the study area. A**: The inlay gives an overview of the tectonic situation in this area, with EP = Eurasian Plate, PH = Philippine Sea Plate, PP = Pacific Plate, NA = North American Plate. **B**: Overview of the Nankai Trough area (modified from [14]), where the Philippine Sea plate subducts beneath the Eurasian plate with a rate of 4.1 to 6.5 cm/yr. Site C0002 is situated at the southern rim of the Kumano forearc basin, which is located between the Muroto Basin in the south-west and the Enshu trough in the north-east.

The SciGas system was again used during IODP NanTroSEIZE Exp. 338, where a borehole was successfully drilled to 2007 meter below seafloor (mbsf) at Site C0002 in the Kumano forearc basin, SE offshore the Kii peninsula (Figure 1A, B). Here we evaluate the scientific value of the current SciGas system (cf. Methods). We analyze samples taken from Hole C0002F drilling mud gas for hydrocarbon gas content with a quadruple mass spectrometer (QMS) and a gas chromatograph (GC). While the QMS only detected methane (hereafter termed C_1-QMS_), the GC allowed identification and quantification of higher hydrocarbons in addition to methane, i.e. C_2_H_6_ and C_3_H_8_ (hereafter termed C_1-GC_, C_2-GC_, C_3-GC_). These concentrations are then compared to shipboard measurements with the gas chromatograph – natural gas analyser (GC-NGA) of the SciGas system and a second shipboard monitoring system provided and operated by Geoservices (cf. Methods). Different gas ratios and temperature estimations using shorebased and shipboard datasets were used as well. Additionally, hydrogen isotopic composition of methane (δD_CH4_) was determined to further elucidate the origin of methane.

The SciGas system is relatively new onboard *D/V Chikyu*, and with increasing importance of the riser drilling technology for future drilling operations, it is necessary to analyse and evaluate the scientific value and limitations of drilling mud gas monitoring. With this work we hope to support and contribute to future studies working with drilling mud gas data obtained onboard *D/V Chikyu.*

### Geological setting

North-west directed subduction of the Philippine Sea plate (PSP) beneath the Eurasian Plate at a rate of ca. 4.1 – 6.5 cm/yr formed the Nankai Trough accretionary complex [15,16]. The northern part of the PSP comprises sediments from the Shikoku Basin, which formed during backarc spreading of the Izu Bonin Arc ca. 23 Ma ago [17]. Subduction and accretion started around 15 Ma, stopped at ca. 12 Ma and continued ca. 6 Ma [18-20].

**Figure 2:**
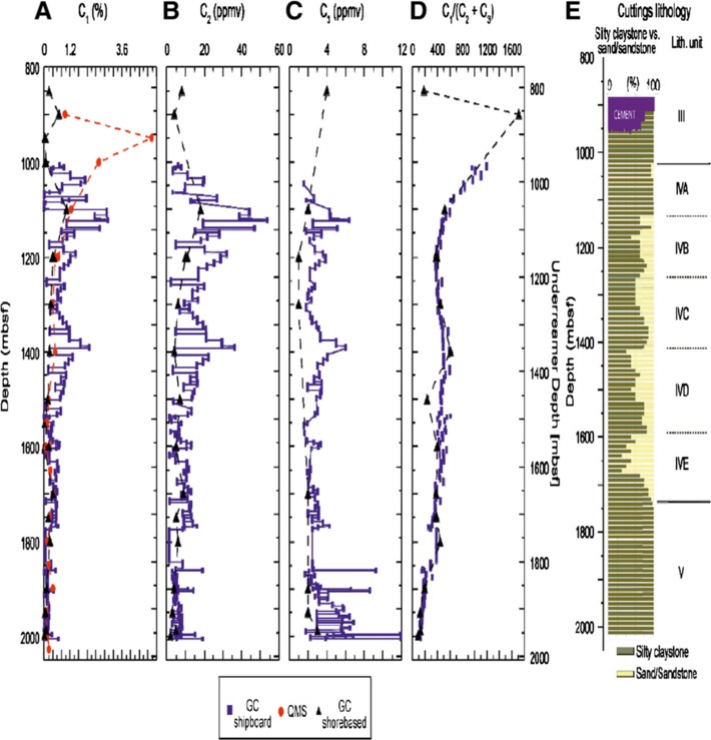
**Results of onshore gas analyses and comparison with shipboard data.** Hydrocarbon gas components in drilling mud gas samples analyzed onshore with a gas chromatograph (GC shorebased; black triangles) and quadruple mass spectrometer (QMS; red dots) compared to shipboard GC-NGA real-time data (GC shipboard, blue squares; [23]). **A**: C_1_ = methane, **B**: C_2_ = ethane, **C**: C_3_ = propane, **D**: (C_1_/(C_2_ + C_3_)) = Bernard parameter). **E**: Lithological column modified from [23]. “Underreamer depth” denotes the position of borehole widening and potential additional gas release caused by the underreamer. Borehole widening took place around 40 m above the borehole depth. See Methods section.

**Figure 3:**
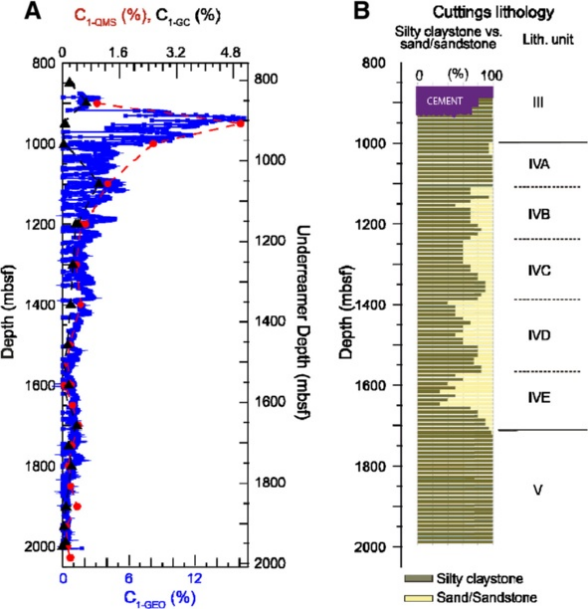
**Results of onshore methane analyses and comparison with shipboard data from Geoservices.** Methane determined by **(A)** the QMS (C_1-QMS_; red dots) and by GC (C_1-GC_, black triangles) compared with shipboard methane data from Geoservices (C_1-Geo_; blue squares and line). Please be aware of the different scales of the individual x-axis at the bottom and at the top. **B**: Lithological column, modified from [23]. “Underreamer depth” denotes the position of borehole widening and potential additional gas release caused by the underreamer. Borehole widening took place around 40 m above the borehole depth. See Methods section.

Site C0002 is situated in the late Miocene Kumano forearc basin, which is the most intensely studied area among the Nankai forearc basins (i.e. from SW to NE: Hyuga basin, Tosa basin, Muroto basin, Kumano basin, Enshu trough; Figure 1B). Around 1.67 Ma ago, sedimentation increased significantly as a consequence of splay fault activity in the accretionary prism [21]. Nowadays, the Kumano basin extends around 100 km from west to east and ca. 80 km from north to south. At Site C0002, the lithology was investigated by drilling and coring 10 boreholes (C0002B, C0002D, C0002F; C0002H, C0002J, C0002K, C0002L, C0002M; C0002N; C0002P; Table 1). The upper ca. 826 m are separated in two units, with Unit I from 0 – 126 mbsf and Unit II from 126 mbsf to 826 mbsf [22]. While both units are dominated by hemipelagic mudstone, intercalations of silty-sandy turbidites and ash layers are more abundant in Unit I. Following Unit II are basal forearc basin sediments (Unit III), which comprise silty claystone with scattered bioturbation and glauconite-rich zones. Further downhole, in Hole C0002F, the upper accretionary prism (Unit IV) was encountered at ca. 1025.5 mbsf [23] (Figures 2 and 3). The boundary was defined based on a pronounced increase in interbedding of sand-, silt- and mudstone [23]. The varying sand content was used to differentiate individual subunits from 1025.5 – 1140.5 mbsf, 1140.5 – 1270.5 mbsf, 1270.5 – 1420.5 mbsf, 1420.5 – 1600.5 mbsf and 1600.5 – 1740.5 mbsf (Table 1). Below 1740.5 mbsf, Unit V starts, which consists mainly of silty to fine silty claystone. In Hole C0002F, starting with Unit IV, all boundaries include an uncertainty of 50 – 70 m, which is caused by the underreamer borehole assembly (BHA) that was used for widening the borehole during riser drilling [23]. In Hole C0002N, the Unit IV/V boundary was already present at 1665.5 mbsf [24]. Unit V could be traced until 3058.5 mbsf in Hole C0002P, which is so far the deepest hole drilled at Site C0002 [24].

**Table 1:** **Overview of depth intervals for units I to V and subunits at Site C0002**

**Unit**	**Subunits**	**C0002B [mbsf]**	**C0002D [mbsf]**	**C0002F [mbsf]**	**C0002H [mbsf]**	**C0002J [mbsf]**	**C0002K [mbsf]**	**C0002L [mbsf]**	**C0002M [mbsf]**	**C0002N [mbsf]**	**C0002P [mbsf]**
I			0 - 135.8								
II		135.8 - 826.3	135.8 - 826.3				200 – 505	200 – 505	475 - 512.5		
III		834.0 - 921.7		875.5 – 1025.5		902 - 926.7				875.5 - 975.5	
IV		921.7 - 1052.5		1025.5 – 1740.5	1100.5 - 1120					975.5 - 1665.5	
	IVA			1025.5 – 1140.5							
	IVB			1140.5 – 1270.5							
	IVC			1270.5 – 1420.5							
	IVD			1420.5 – 1600.5							
	IVE			1600.5 – 1740.5							
V				1740.5 – 2004.5						1665.5 - 2325.5	1965.5 - 3058.5

Overview of depth intervals for Units I to V and subunits at Site C0002.

## Results

### Results of shipboard analyses

**Figure 4:**
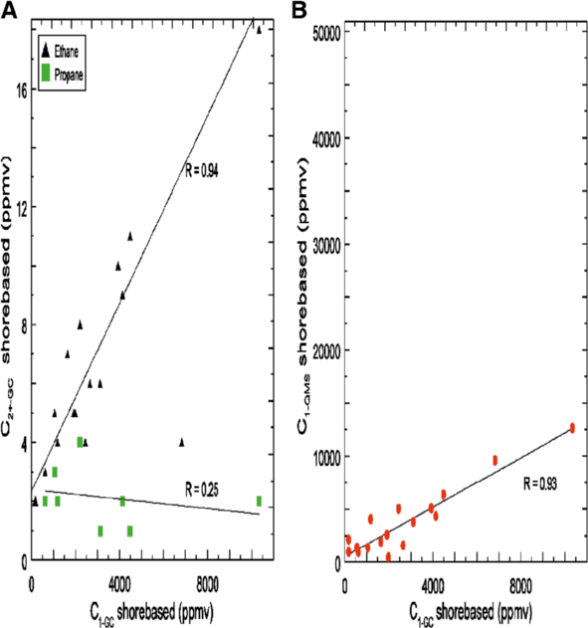
**Correlation of methane with higher homologues. A**: Methane (C_1-GC_) vs. higher hydrocarbon components (C_2+−GC;_ ethane: black triangle; propane: green square), all determined by shorebased analyses with a gas chromatograph. **B**: Methane following shorebased analyses with a gas chromatograph (C_1-GC_) vs. methane following shorebased analyses with a quadruple mass spectrometer (C_1-QMS_).

**Figure 5:**
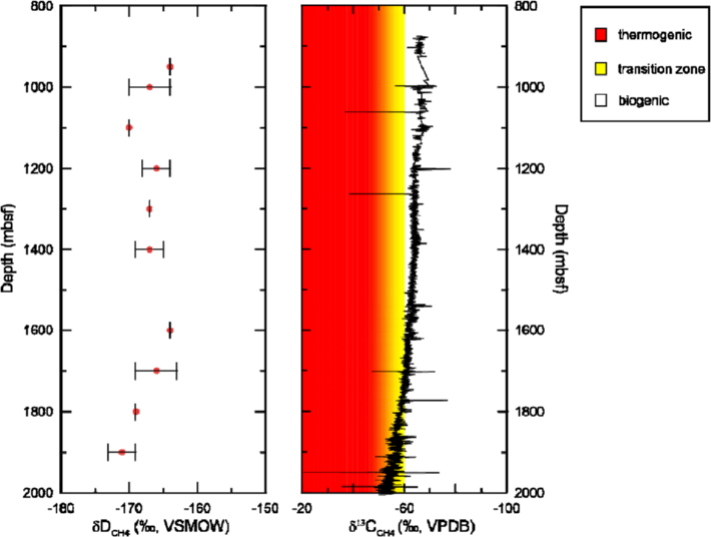
**Results for deuterium and methane carbon isotope ratios.** Left panel: δD_CH4_ values determined for different gas samples from IODP Expedition 338 [23]. Horizontal bars indicate the standard deviation of duplicate measurements. Right panel: δ^13^C_CH4_ data obtained during drilling borehole C0002F (modified from [23]). Starting at 1700 mbsf, the δ^13^C_CH4_ data indicate a shift to methane of thermogenic origin (boundaries after [26]).

The results of the measurements are displayed in Figures 2, 3, 4 and 5. The results of the shipboard measurements are comprehensively documented in [23], and will only be reviewed briefly. Both the GC dataset from the SciGas system and the Geoservices dataset were dominated by C_1_, with concentrations of up to 8.64% and 16.4%, respectively (Figure 2A; [23]). C_2_ and C_3_ were only found in minor concentrations, with up to 0.03% and 0.09% for C_2_, and 0.013% and 0.23% for C_3_, in the Geoservices and GC datasets, respectively (Figure 2B, C; [23]). Shipboard values for δ^13^C_CH4_ stayed below – 60‰ at depths shallower than 1700 mbsf, and gradually increase farther downhole (Figure 5). Between 900 and 1000 mbsf, methane concentrations above 1% were encountered, which caused a malfunction of the methane carbon isotope analyzer onboard *D/V Chikyu* [25]. As a consequence, no accurate measurements were possible, and the data were interpolated (Figure 5).

### Results of shorebased analyses

With values between 0.01 and 1.04% in the GC data and 0.04 and 4.98% in the QMS data, C_1_ was also the dominant gas species in the shorebased data, followed by C_2-GC_ (2 – 18 ppmv) and C_3-GC_ (2 – 4 ppmv) (Table 2). Higher homologues were not detected.

**Table 2:** **Results for shore-based analysis of drilling mud gas samples**

**Sample ID 338-C0002F-00**	**Sampling vial**	**Total depth (mBRT)**	**lag depth (mbsf)**	**C**_**1-QMS**_ **(ppm)**	**C**_**1-GC**_ **(ppm)**	**C**_**2-GC**_ **(ppm)**	**C**_**3-GC**_ **(ppm)**	**δD**_**CH4**_ **(‰, VSMOW)**
244GMW-WR	Glass	2817.5	850	NA	2220	8	4	NA
013GMW-WR	Glass	2867.5	900	9610	6830	4	BD	BD
023GMW-WR	Glass	2917.5	950	49800	420	BD	BD	−164
033GMW-WR	Glass	2967.5	1000	25300	96	BD	BD	−167
033GMW-WR	Iso	2967.5	1000	NA	690	BD	BD	NA
070GMW-WR	Glass	3067.5	1100	12600	10350	18	2	−170
079GMW-WR	Glass	3167.5	1200	6360	4490	11	1	NA
079GMW-WR	Iso	3167.5	1200	5080	3940	10	BD	−166
128GMW-WR	Glass	3267.5	1300	3800	3120	6	1	−167
129GMW-WR	Iso	3367.5	1400	5040	2460	4	BD	−167
146GMW-WR	Glass	3467.5	1500	1940	1650	7	BD	NA
180GMW-WR	Glass	3517.5	1550	1380	570	BD	BD	ND
181GMW-WR	Glass	3567.5	1600	2300	440	2	3	NA
181GMW-WR	Iso	3567.5	1600	440	2000	5	BD	−164
197GMW-WR	Iso event	3572.3	1604.8	960	190	BD	BD	BD
225GMW-WR	Glass	3667.5	1700	4340	4140	9	2	−166
226GMW-WR	Glass	3717.5	1750	2580	1930	5	BD	NA
243GMW-WR	Glass	3767.5	1800	1580	2670	6	BD	−169
263GMW-WR	Iso	3767.5	1800	1890	43	BD	BD	NA
277GMW-WR	Glass	3867.5	1900	4050	1200	4	2	−171
278GMW-WR	Glass	3917.5	1950	950	630	3	2	NA
305GMW-WR	Iso	3955	1987.5	1360	1060	5	3	−166
391GMW-WR	Glass	3965.7	1998.2	2140	184	2	BD	NA

Results for shore-based analysis of drilling mud gas samples (iso = Isotube, glass = glass flask, iso event = event gas sampled with Isotube). Total depth is the depth of the borehole when the mud gas was sampled. Lag depth is the sampled depth corrected for the time the drilling mud needs to circulate from the drill bit to the ship. NA denotes “not available”, and was used for samples where the gas concentration in the sample vials was too low for further measurements; BD = below detection limit, mBRT = meter below rotary table; mbsf = meter below seafloor.

Although concentrations of C_2-GC_ are up to four orders of magnitude smaller compared to the concentrations of C_1-GC_, both components show a similar distribution with depth (Figure 2) and are positively correlated with R = 0.94 (Figure 4). The highest values for C_1-GC_ and C_2-GC_ were found at 1100 mbsf, with 1.03% and 18 ppmv, respectively. Both components experience an overall decrease downhole. C_1-GC_ is hardly correlated with C_3-GC_ (R = 0.25), but shows a slightly negative trend (Figure 2) with minor variations downhole. After C_3-GC_ decreases from 4 to 1 ppmv between 850 and 1200 msbf, it slightly increases again with depth to 3 ppmv.

C_1-QMS_ shows a positive correlation with C_1-GC_ (R = 0.93), and at depths greater 1000 mbsf, both C_1-QC_ and C_1-QMS_ experience similar variations and concentrations with depth (Figure 2A, B). In the depth interval between 900 and 1000 mbsf, however, C_1-QMS_ dominates the gas show with almost 5%, whereas C_1-GC_ decreases to 0.42 ppmv (Figure 4B). This deviation might result from gas depletion in the sample vial after the initial total gas analyses carried out with the QMS. The Bernard parameter (i.e. C_1_/(C_2_ + C_3_), [27]) varies between 1708 at 900 mbsf and 92 at 1998.2 mbsf, indicating a relative increase in thermogenic components downhole.

The hydrogen isotopic composition of CH_4_ was uniform; δD-values ranged from -171‰ to -164‰ and averaged around -167 ± 2‰ (Figure 5). The standard deviation of duplicate measurements was on average 2‰.

### Comparison between shipboard and shorebased drilling mud gas data

A comparison between shorebased GC, QMS and onboard GC-NGA and Geoservices data [23] is shown in Figures 2 and 3, and Additional file 1: Figures S1 and S2 in the supplementary material). Above 1300 mbsf, drilling mud gas monitoring by Geoservices showed higher methane and ethane concentrations than GC and QMS (C_1-GC_, C_1-QMS,_ both on the upper x-axis in Figure 3; C_2-GC_ in Figure 2B). Below 1300 mbsf, concentrations correspond to the real-time values (Figures 2A and 3, Additional file 1: Figure S1). The small data coverage for C_3-GC_ prevents a thorough comparison, and adds to the low correlation in Additional file 1: Figure S1F, but the available shorebased and shipboard C_3_ concentrations are in the same order of magnitude.

Though shorebased GC-measurements yielded lower methane concentrations than shipboard mud-gas monitoring by SciGas and Geoservices, overall trends are similar in all three data sets (Figure 2A-D; Additional file 1: Figure S1A-D). Ethane and propane, however, show no clear correlation (Additional file 1: Figure S1E, F). By contrast, the Bernard parameter based on online GC-NGA data corresponds with the Bernard parameter from shorebased measurements (Figure 2D, Additional file 1: Figure S1G). Except for an outlier at 1500 mbsf, relative changes in the gas composition are well reproduced by onshore measurements, and give the same qualitative estimation of thermal maturity (Figure 2D).

A comparison between the C_1_/C_2_, C_1_/C_3_ and C_2_/C_3_ ratios of the shipboard (GC-NGA only) and shorebased measurements is given in the supplementary material (Additional file 1: Figure S2). Shipboard and shorebased datasets are well correlated for C_1_/C_2_ ratios, (R = 0.87), but deviate considerably from each other with respect to C_1_/C_3_ (R = 0.36) and C_2_/C_3_ ratios (R = 0.41).

Similar findings arise when comparing the Geoservices data with the shorebased GC data (Figure 3A). While the C_1_/C_2_ ratios correspond well with a correlation coefficient of R = 0.95 (by disregarding the single outlier), C_2_/C_3_ ratios have a negative correlation with R = −0.5, and C_1_/C_3_ ratios show data scatter with R = 0.16 (see Additional file 1: Figure S3 in the supplementary material).

## Discussion

### Technical considerations

During IODP Exp. 338, the mud-gas monitoring system from Geoservices recorded distinctly higher absolute hydrocarbon gas concentrations than the recently installed SciGas system [23]. The lower gas recovery of the latter are likely due to the technical configuration [25] that can only be adjusted and optimized during riser drilling operations. As a consequence, relative changes are less pronounced, and differentiation between formation- and/or drilling-related artefacts is more difficult [1]. In addition, the comparison of data sets that result from analyses of discrete samples and continuous on-line monitoring, respectively, is complicated by the synchronization of measurements. This is particularly true for analyses with long run-times. For example, GC-NGA analysis require 20 minutes [25] and, with drilling proceeding at an average rate of 30 m penetration per hour, interpolate over a corresponding depth range of 10 m. This issue becomes obvious in the low correlation of higher hydrocarbon gases in shorebased and shipboard datasets (see Additional file 1: Figure S1E for ethane and Additional file 1: Figure S1F for propane concentrations, Additional file 1: Figure S2A-C for C_1_/C_2_, C_2_/C_3_, and C_1_/C_3_ ratios). For C_2_ and C_3_, concentrations differed significantly between the depths where the gas was sampled for onshore analyses and where the last shipboard measurement took place. For C_1_, the problems were of minor importance, because concentrations were high and relatively stable.

Ratios of hydrocarbon gases are commonly used to evaluate relative variations, e.g. pixler plots [28], star/spider diagrams [1,29,30] and parameters such as hydrocarbon wetness, balance and character [31]. Most of these methods require reliable estimations of higher hydrocarbons (i.e. C_5+_), which cannot be derived by conventional degassing methods due to inefficient liberation of higher hydrocarbons. At in situ temperature and pressure, individual gaseous components can be in solution with the drilling fluid and/or the seawater (e.g. [32-34]), and the solubility increases with increasing number of hydrocarbon gases [32]. During ascend of the drilling mud, pressure and temperature decrease, which reduces the solubility of the hydrocarbon gases, and increases the potential to be extracted by the degasser. However, due to their boiling points < 0°C, only C_1_ to C_4_ remain in gas phase at atmospheric conditions. Efficient extraction of C_5+_ requires heating of the drilling mud during degassing. Although such instruments exist (e.g., FLAIR, see [1]) none were available during IODP Expedition 338 [25]. Additionally, our results suggest that simple gas-to-gas ratios are not sufficient to orderly evaluate the SciGas system (Additional file 1: Figures S2 and S3; Additional file 2: Table S1). The relatively good correlation of R = 0.95 with the Geoservices system (Additional file 1: Figure S3A) with respect to the C_1_/C_2_ ratios is contrasted with a bad correspondence of C_2_/C_3_ and C_1_/C_3_ ratios, and thus, the comparison is ambiguous (Additional file 1: Figure S3B, C). Therefore, for a qualitative evaluation, we focus also on the Bernard parameter [27] and the hydrocarbon wetness, which is expressed as total wet gas fraction (TWG), i.e. (∑ C_2_ + C_3_)/(∑ C_1_ – C_3_) × 100) [27,31,35-37]. At a TWG ≤ 5.0%, the gas composition is dominated by methane, either because temperature and time were insufficient to produce higher-order hydrocarbons, or the hydrocarbons are overmature [38]. Our samples show a TWG of ≤ 1.15% and are in good agreement with the TWG derived from shipboard GC-NGA measurements (supplementary material, Additional file 2: Table S1). TWG for the Geoservices data is, with up to 2.4%, more than twice as high as the shorebased GC data, but still below the 5.0% threshold. At depths > 1950 mbsf, all three datasets show an increase in TWG (supplementary material, Additional file 2: Table S1). Individual TWG values imply a good correlation (see supplementary material, Additional file 1: Figure S4A, R = 0.81, Additional file 1: Figure S4B, R = 0.95) and all three datasets confirm the overall absence of wet gas, despite being acquired by different instruments.

**Figure 6:**
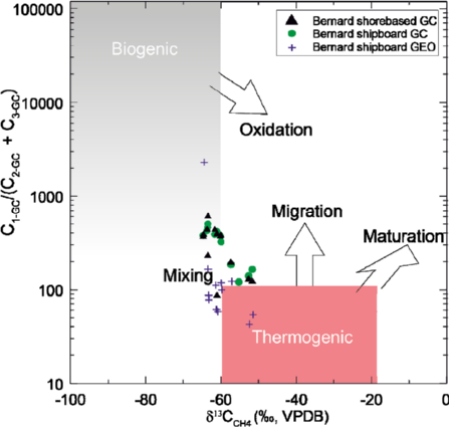
**Qualitative estimation of gas mixing.** Bernard diagram based on the Bernard parameter (based on shipboard and shorebased data) and the shipboard δ^13^C_CH4_ data (after [27]). All three datasets clearly plot in the mixing regime.

When comparing the different Bernard parameters (i.e. C_1_/(C_2_ + C_3_), [27]) in a Bernard diagram (i.e. Bernard parameter vs. δ^13^C_CH4_, Figure 6), both shipboard and shorebased GC measurements are again in good agreement. In contrast, Geoservices data show larger scatter and a relatively higher amount of ethane and propane. Also, for two samples from depths > 1950 mbsf, only Geoservices samples plot in the thermogenic regime (Figure 6). Simultaneously, plotting δD_CH4_ vs. δ^13^C_CH4_, following the classification of [39] clearly indicates microbial CO_2_ reduction to be responsible for methanogenesis at depths shallower than 1600 mbsf (Additional file 1: Figure S4 in the supplementary material). Similar results were derived during riser drilling at Site C0009, which is around 15 km north of Site C0002 [12]. At Site C0009, drilling mud gas monitoring revealed the predominance of biogenic methane to the bottom of the borehole at ca. 1600 mbsf. The prevalence of carbonate reduction implies that the drilled depth intervals at Site C0002 and C0009 are sulfate free zones, and that sulfate reducing bacteria are absent (e.g., [39,40]). Moreover, methanogenic CO_2_ reduction is a common process in marine environments, while C_1_ has higher δ^13^C_CH4_ and lower δD_CH4_ values in freshwater [39]. At Site C0002, thermogenic gas does not occur earlier than in the deepest sample (i.e. at 1987.5 mbsf), which supports the data by Geoservices (Figure 6).

Following the results for the TWG and the Bernard parameter, the SciGas as well as the Geoservices datasets allow us to qualitatively evaluate the gas composition despite the different degassing systems. Nonetheless, both the TWG and the Bernard parameter point to generally higher ethane and propane concentrations using the Geoservices degassing system (Figure 6, Additional file 1: Figure S5; Additional file 2: Table S1). Reasons for the underestimation of ethane and propane with the SciGas system are manifold. Most likely, the configuration of the SciGas degasser caused insufficient gas liberation from the drilling mud [25]. At times of low mud pump activity, the mud level in the flow line declined. Contrary to the degassing system from Geoservices, it was impossible to adjust the SciGas degasser in height in real-time. This caused insufficient stirring of the drilling mud, thus the gas phase was less efficiently liberated and more likely to be contaminated by atmospheric gases. Consequently, the SciGas only detected concentrated gases, which degass readily without further stimulation.

### Origin of gases

At depths greater 1950 mbsf, the Bernard parameter, the δD-δ^13^C_CH4_ plot and the TWG ratios point to a relative increase in thermogenic gases (Figures 5 and 6, Additional file 1: Figure S4). At the same time, no wet gas composed of C_2+_ > 5% (i.e., > 50000 ppmv; e.g., [38]) was encountered (Figures 2 and 3; Table 1). Wet gas is generated at elevated thermal maturity, which can be evaluated using vitrinite reflectance R_o_ ([26], and references therein). The latter is estimated by the shipboard δ^13^C_CH4_ values using the empirical relationship δ^13^C_CH4_ (‰) = 15.4 log_10_ %R_o_ - 41.3 ([26], and references therein). Computing the vitrinite reflectance led to values below 0.6, i.e. below a maturation indicative for the onset of the oil and gas window (supplementary material, Additional file 2: Table S1).

**Figure 7:**
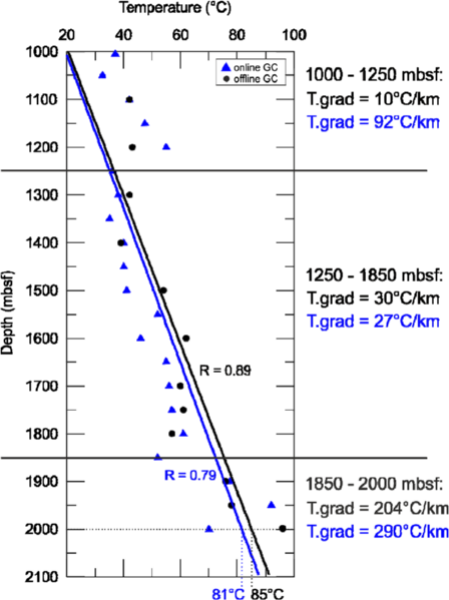
**Temperature estimation for in situ conditions.** Temperature estimation following the C_1_/C_2_-Temperature-TOC relationship shown in Additional file 1: Figure S6 in the supplementary material. Geothermal gradients are similar, with 39.3°C and 42.5°C for the shipboard and shorebased data, respectively.

In general, thermal maturity is influenced by the geothermal gradient and the time available for maturation [41,42]. For the Kumano forearc basin, following the heat flow determination of [43], the geothermal gradient is estimated to be ca. 40°C/km. This gradient was already corrected for sedimentation rate and subsidence [43]. Giving a bottom water temperature of 2.4°C at Site C0002 [43], an *in situ* temperature of 82.4°C can be concluded for a thermal conductivity of 1.5 W m^−1^ K^−1^ at a depth of 2000 mbsf. This estimate is confirmed by using an empirically determined C_1_/C_2_-TOC-temperature relationship originally compiled for safety purposes by [44] (Additional file 1: Figure S6 in the supplementary material). Plotting TOC data from Expedition 338 [23] and the C_1_/C_2_ ratios based on the shorebased and shipboard GC measurements shows a rather large scatter (Additional file 1: Figure S6 in the supplementary material), which is understandable giving the uncertainties in drilling mud gas monitoring and the limitations of the empirical C_1_/C_2_-TOC-temperature relationship [44]. Nonetheless, a general increase in the temperature with depth can be observed, with 2 remarkable changes in trend at 1250 mbsf and 1850 mbsf (Figure 7). These depths do not correlate with any of the lithological boundaries (Table 1). Below 1850 mbsf, the temperature gradients are unreasonably high, which is most likely due to the small number of data points, and the increasing influence of migrated thermogenic hydrocarbon gas.

In general, the C_1_/C_2_-TOC-temperature relationship is based on the assumption that the hydrocarbon gases were produced *in situ* [44]. Therefore, migration of thermogenic gases and mixing with hydrocarbon gases that were produced *in situ* easily compromises the interpretation derived by the C_1_/C_2_-TOC-temperature relationship. At the same time, the temperature estimate is easily influenced by the degasser configuration and the drilling operation, mainly due to the selective detection of C_2_ (cf. section “Technical Considerations”). For these reasons and given the small amount of data points, it might be misleading to discuss the individual temperature gradients separately. Applying a simple linear fit to both datasets points to an *in situ* temperature between 81°C and 85°C (Figure 7) at 2000 mbsf, which is in agreement with the estimations provided by [43]. Although hydrocarbon generation already starts at 50°C, higher hydrocarbons are usually encountered at temperatures > 100°C (e.g., [45]).

At the same time, in addition to the low temperature, the geological timescale for hydrocarbon generation at these temperatures was probably too short. A simple quantitative measure for the time-temperature-related maturity is the time-temperature index (TTI) [41,42], which can be calculated as follows:

1 $$ \mathrm{T}\mathrm{T}\mathrm{I} = {\displaystyle \sum \left({\updelta \mathrm{t}}_{\mathrm{i}}\right)\ \left({{\mathrm{r}}_{\mathrm{i}}}^{\mathrm{n}}\right)} $$

where δt_i_ denotes the time interval spent in the i-th 10°C temperature interval, and r_i_^n^ is the temperature factor related to the individual temperature interval [41]. The TTI is based on the assumption that the rate of the chemical reaction responsible for hydrocarbon generation from kerogen doubles for every interval of 10°C, while variations in kerogen composition are neglected [46]. In addition, we make the following assumptions: (i) the material at ca. 2000 mbsf at Site C0002 is similar to the material encountered in Unit III at NanTroSEIZE Sites C0011 and C0012, and thus, has a maximum age of 9 Ma (Expedition 348 Scientists, in preparation); (ii) if the material was deposited at 9 Ma, than it falls in the period where subduction ceased and thus, it was only subject to diagenesis between 9 and 6 Ma [20] (iii) following [47], we assume that the material was not buried deeper than 100 mbsf, and thus, the material experienced temperatures of up to 3°C (based on a temperature gradient of ca. 100°C/km, and a seafloor temperature of ca. 2°C; [47,48] (iv) the material was subducted at 6 Ma [47], and (v) experienced an increase in temperature by 79°C, leading to 82°C at 2000 mbsf based on the work of [43]. These estimates result in two different heating rates: a pre-subduction heating rate of 2.3°C/Ma and a post-subduction heating rate of 20°C/Ma. Based on these rates, we calculated a cumulative TTI of 0.23 (Table 3). This value is below the onset of petroleum production (TTI ≥ 15), and far too low to indicate wet gas (TTI ≥ 1500) [42].

**Table 3:** **Temperature and time intervals for calculating the time-temperature index**

**Temp. interval (°C)**	**r** ^**n**^	**δt (Ma)**	**Interval TTI**	**Total TTI**
0 - 10	2^−10^	0.53	0.0005	0.0005
10 - 20	2^−9^	0.76	0.0015	0.0020
20 - 30	2^−8^	0.76	0.0030	0.0050
30 - 40	2^−7^	0.76	0.0059	0.0109
40 - 50	2^−6^	0.76	0.0119	0.0228
50 - 60	2^−5^	0.76	0.0237	0.0465
60 - 70	2^−4^	0.76	0.0475	0.0940
70 - 80	2^−3^	0.76	0.0949	0.1889
80 - 82	2^−2^	0.15	0.0380	0.2269

Overview of the different temperature and time intervals for which a time-temperature index (TTI) was calculated. “Interval TTI” denotes the TTI for an individual temperature interval, whereas “cumulative TTI” is the sum of the interval TTIs. See text for explanations.

Consequently, following our estimations for R_0_, *in situ* temperature and the TTI, *in situ* production of significant amounts of higher hydrocarbons is unlikely at depths < 2000 mbsf. Beside *in situ* production, gases tend to follow the pressure gradient and migrate in adjacent rocks along faults or fractures, or via inter-granular diffusion. For shallower gas accumulations, this can lead to mixing of biogenic and thermogenic gas. The TWG allows first qualitative estimations, but identifying secondary effects such as mixing remains difficult. Indeed, the Bernard diagram points to a gas composition, which is affected by mixing rather than showing a clear biogenic or thermogenic signal (Figure 6). Mixing can occur in different ways, either due to active gas migration along faults or fractures, or the gas mixture was derived by diffusive migration leading to isotope fractionation [49]. For methane, diffusive migration would lead to an enrichment of the light carbon isotope, and thus, the migrated gas would plot in the biogenic or mixed regime despite being derived from a thermogenic source [49].

At Site C0002, a fault zone is indicated between 1500 and 1640 mbsf based on resistivity data obtained during logging-while-drilling [23]. This corresponds to an increase in TWG of the Geoservices and the shorebased dataset at 1600 mbsf, pointing to active or recently active migration of higher hydrocarbons from greater depths and subsequent mixing (Additional file 2: Table S1). Detailed analyses of possible gas migration and mixing will be covered in future studies evaluating data from the recently finished IODP Exp. 348 using noble gas isotopes from Holes C0002F and C0002N.

## Conclusions

In conclusion, shipboard and shorebased analyses allow the same qualitative estimation about the genetic origin of the drilling mud gas. Differences in absolute concentrations of the SciGas and Geoservices degassing systems are most likely caused by the different configurations of the individual degassers, which led to an underestimation of higher hydrocarbons when using the SciGas system. Comparison of the individual datasets by simple gas ratio analysis was ambiguous, therefore we chose the Bernard parameter and the total wet gas ratio to qualitatively analyze and compare the individual datasets. Eventually, we showed that, beside the technical problems encountered during IODP Exp. 338, the SciGas system produced reliable data, which helped to qualitatively estimate temperature, maturity, and possible mixing of the hydrocarbon gases. We found that microbial methane was present to up to 1600 mbsf, with thermogenic gas production probably not starting at depths shallower 2000 mbsf. Consequently, the SciGas system onboard *D/V Chikyu* is suitable for detecting qualitative changes, and allows a first estimation of the contribution of biogenic and thermogenic hydrocarbon gas.

## Methods

### Shipboard analyses

**Figure 8:**
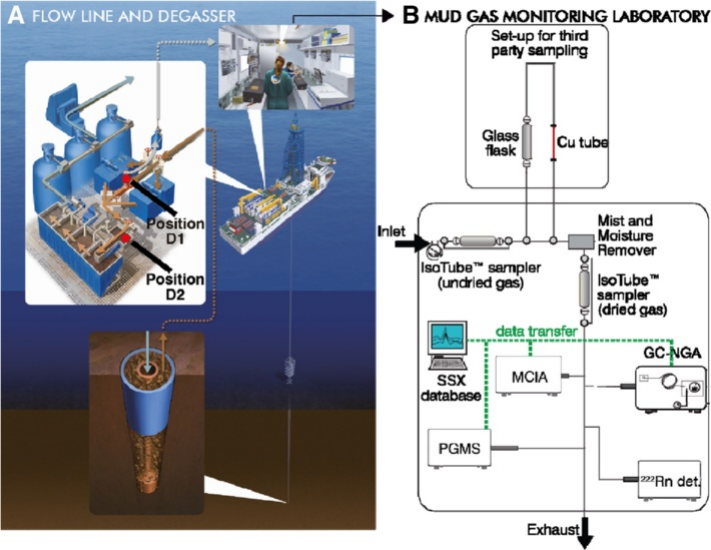
**Schematic of the drilling mud gas monitoring set-up onboard D/V Chikyu.** Drilling mud gas monitoring set-up during IODP Exp. 338 (modified from [25]; 3D schematic © JAMSTEC). “Position D1” denotes the position of the SciGas degasser, whereas “Position D2” indicates the position of the Geoservices degasser. Sampling was either possible with the onboard IsoTube sampling system, or with a third-party sampling line including glass flask and Cu tubes. Prior to onboard measurements, the drilling mud gas was dried with a mist and moisture remover. The results of the measurements were stored in the onboard SSX datasystem.

Generation of shipboard data is comprehensively explained in Expedition 338 Scientists [25] and in the following, will be briefly summarized (Figure 8). Shipboard data were compiled in real-time by the SciGas and Geoservices systems, of which each uses an individual degasser to extract gas from the drilling mud. Compared to the SciGas system (Figure 8, position D1), the degasser from Geoservices was placed further downstream the flow line (Figure 8, position D2) and was adjustable in height in case the mud level dropped. This instrument provides an agitator stirring the mud to maximize separation of the gas phase from the fluid phase. Afterwards, the gas travelled through a ca. 50 m long PVC tubing with ca. 3 mm inner diameter, causing a time difference of ca. 6 minutes between gas extraction and arrival at the mud gas monitoring laboratories. At the Geoservices mud logging laboratory, total gas concentration and gas composition were determined separately using two individual gas chromatographs. Prior to measurements, the drilling mud gas had to pass a mist and moisture remover (i.e. a “dehydrator”). Contamination checks were carried out during IODP Expedition 338, but no signs for contamination at the mist and moisture remover were found [25].

While position and configuration of the degasser of the SciGas system precluded any height adjustment (Figure 8, position D1), the extracted drilling mud gas was subject to a broader range of measurements. After having bypassed the sampling line, the gas was dried with a mist and moisture remover [25]. For δ^13^C_CH4_ analysis isotope fractionation potentially caused by the mist and moisture remover is negligible [13]. Afterwards, the gas composition was first analyzed by a methane carbon isotope analyzer (MCIA), followed by a GC-natural gas analyzer (GC-NGA), a detector that counts radioactive decay of radon, and a process gas mass spectrometer (PGMS) (for detailed information about the instruments and measurement specifications, please see [25]).

In this manuscript, shipboard data used for further evaluation of and comparison with the shorebased data include the MCIA and GC-NGA datasets. Precision (expressed as relative standard deviation, RSD) of measurements with the MCIA and GC-NGA were 0.4% and 1.4 – 1.5% RSD, respectively. The data produced by Geoservices will be included as well to highlight the differences of the Geoservices and SciGas monitoring systems. δ^13^C_CH4_ values are reported in notion to the Vienna Peedee belemnite (VPDB) standard in parts per mil (‰) [25].

### Drilling Mud Gas sampling and analyses

During IODP Exp. 338, before being analyzed by onboard instruments, the gas phase flowed through a third-party sampling line and the onboard Isotube sampling system [25]. Sampling took place between 850 and 1998.5 mbsf using glass flasks and copper (Cu) tubes for the third-party flow line, and Isotubes for the Isotube system (Table 2). All samples were taken before the gas passed the mist and moisture remover (Figure 8).

In total, 23 of the drilling mud gas samples collected during IODP Exp. 338 [23] were subject to shore-based analyses by the QMS (Pfeiffer Omnistar) and the GC (SRI-8610) equipped with a Haysep D column and a flame ionization detector. Detection of methane with the QMS is often subject to isobaric interference with ^16^O, therefore we focused on m/z = 15. The signal strength was still 85%, which allowed the measurement of relative changes in the gas concentrations. For both the QMS and the GC, the detection limit for hydrocarbon gases was set to 1 parts per million by volume (ppmv). The relative errors for the QMS and QC measurements are listed in Additional file 3: Table S2.

### Stable hydrogen isotope analysis of methane

Out of the 23 samples taken, 14 were subject to hydrogen isotope analysis. Methane concentrations were high enough for reliable stable hydrogen isotope analysis in 12 of the 14 samples taken (Table 2). Prior to the analysis, samples were given time to adjust to room temperature. The stable hydrogen isotopic composition of CH_4_ was analyzed by isotope ratio monitoring gas chromatography/mass spectrometery (irm-GC/MS) using a Thermo Finnigan Trace Ultra GC, connected to a Thermo Finnigan DELTA V Plus mass spectrometer via Thermo Finnigan GC-Isolink interface as reported previously [13]. The analysis involved online transfer of samples from a high temperature conversion reactor (containing an empty ceramic tube covered with graphite layer that was kept at a temperature of 1440°C) in which compounds were pyrolyzed to molecular hydrogen, carbon, and carbon monoxide, prior to their transfer into the mass spectrometer via Conflow IV interface. The Trace Ultra GC was equipped with a Carboxen column (30 m length, 0.32 mm inner diameter). The carrier gas was helium (1.2 mL min^−1^), the split ratio 1:8, and the temperature of the GC oven and injector were 60°C (isotherm) and 200°C, respectively. The primary standardization of the DELTA V Plus was based on multiple (three to six) injections of reference H_2_ from a lab tank (*δ*D = -96.4 ± 0.3‰ vs VSMOW, 3.2 ± 0.3 V at m/z 2) at the beginning and end of the analysis of each sample. Lab tank H_2_ was calibrated against the certified CH_4_ standard T-iso2 (2.5 vol% CH_4_ in a balance of dry, synthetic air; *δ*^13^C_CH4_ = -38.3 ± 0.2‰ vs VPDB; *δ*D_CH4_ = −138‰ vs VSMOW). We assessed the precision of our analysis by repeated analysis of the standard. The precision was better than 2‰ (1σ). Stable hydrogen isotope analysis of methane requires a peak amplitude of 1 V or higher at m/z 2. Depending on CH_4_ concentration, ~300 μL to ~3000 μL of sample were injected per analysis. All analyses were carried out in duplicate.

Stable hydrogen isotope ratios are reported in *δ*D notation (per mil, ‰) relative to the Vienna Standard Mean Ocean Water (VSMOW), with *δ*D = [(R_sample_-R_VSMOW_)/R_VSMOW_] · 10^3^, where R = ^2^H/^1^H and R_VSMOW_ = (155.76 ± 0.05) × 10^−6^ [50].

## Additional files

Additional file 1: Figure S1. – Correlation of SciGas shipboard and shorebased gas ratios. While the (A) C_1_/C_2_ ratios and the (D) Bernard parameters show a good correspondence, the data scatter of the (B) C_1_/C_3_ ratios and (C) C_2_/C_3_ ratios preclude any clear correlation. **Figure S2.** –Correlation of SciGas shipboard and shorebased gas ratios. While the (A) C_1_/C_2_ ratios and the (D) Bernard parameters show a good correspondence, the data scatter of the (B) C_1_/C_3_ ratios and (C) C_2_/C_3_ ratios preclude any clear correlation. **Figure S3.** –Correlation of SciGas shipboard and shorebased data with the dataset produced by Geoservices (“GEO”) during Exp. 338 [23]. Shown are (A, D) C_1_/C_2_, (B, E) C_2_/C_3_ and (C, F) C_1_/C_3_ ratios. “Shipboard” refers to data obtained simultaneously with Geoservices data. “Shorebased” refers to data gained by onshore analyses of samples taken from the SciGas system during IODP Exp. 338. **Figure S4.** – Shorebased δD_CH4_ values plotted against shipboard δ^13^C_CH4_ data (diagram modified from [26]). The sampled methane is derived by microbial carbonate reduction. The values point to a contribution of thermogenic sources at depth. **Figure S5.** –Correlation between the different total wet gas ratios (TWG). (A) The panel shows a relatively good correlation of the shorebased SciGas data with the data from Geoservices (blue circles, R = 0.81), while the two shipboard datasets reveal a larger scatter (triangles; R = 0.46). (B) Neglecting the outlier at ca. 1600 mbsf, the total wet gas ratios of both GC datasets correspond well with R = 0.98. **Figure S6.** –C_1_/C_2_-Temperature-TOC diagram following the empirical relationship compiled by JOIDES PPSP [44]. Shipboard TOC (= total organic carbon) data was used to associate the SciGas shipboard (white points; [23]) and the onshore GC data (blue points) to temperatures. Temperature estimations and comprehensive explanations are given in Figure 7 in the main text. Additional file 2: Table S1. Overview of the different gas ratios. GEO = shipboard data acquired by Geoservices, GC = data produced using a gas chromatograph, TWG = total wet gas ratio, R_0_ = vitrinite reflectance. Additional file 3: Table S2. Precisions for shorebased QMS and GC measurements. Please be aware that GC precision is given in relative (%) and absolute (ppmv) numbers.
