# Supplementary material for: Real-time drilling mud gas monitoring for qualitative evaluation of hydrocarbon gas composition during deep sea drilling in the Nankai Trough Kumano Basin
Source: Geochem Trans. 2014 Dec 16;15:15. doi: 10.1186/s12932-014-0015-8 (PMC4302130; doi:10.1186/s12932-014-0015-8)
Supplement: Additional file 1: Figure S1. — – Correlation of SciGas shipboard and shorebased gas ratios. While the (A) C1/C2 ratios and the (D) Bernard parameters show a good correspondence, the data scatter of the (B) C1/C3 ratios and (C) C2/C3 ratios preclude any clear correlation. Figure S2. –Correlation of SciGas shipboard and shorebased gas ratios. While the (A) C1/C2 ratios and the (D) Bernard parameters show a good correspondence, the data scatter of the (B) C1/C3 ratios and (C) C2/C3 ratios preclude any clear correlation. Figure S3. –Correlation of SciGas shipboard and shorebased data with the dataset produced by Geoservices (“GEO”) during Exp. 338 [23]. Shown are (A, D) C1/C2, (B, E) C2/C3 and (C, F) C1/C3 ratios. “Shipboard” refers to data obtained simultaneously with Geoservices data. “Shorebased” refers to data gained by onshore analyses of samples taken from the SciGas system during IODP Exp. 338. Figure S4. – Shorebased δDCH4 values plotted against shipboard δ13CCH4 data (diagram modified from [26]). The sampled methane is derived by microbial carbonate reduction. The values point to a contribution of thermogenic sources at depth. Figure S5. –Correlation between the different total wet gas ratios (TWG). (A) The panel shows a relatively good correlation of the shorebased SciGas data with the data from Geoservices (blue circles, R = 0.81), while the two shipboard datasets reveal a larger scatter (triangles; R = 0.46). (B) Neglecting the outlier at ca. 1600 mbsf, the total wet gas ratios of both GC datasets correspond well with R = 0.98. Figure S6. –C1/C2-Temperature-TOC diagram following the empirical relationship compiled by JOIDES PPSP [44]. Shipboard TOC (= total organic carbon) data was used to associate the SciGas shipboard (white points; [23]) and the onshore GC data (blue points) to temperatures. Temperature estimations and comprehensive explanations are given in Figure 7 in the main text. [file 12932_2014_15_MOESM1_ESM.pdf]

Supplementary Material for the manuscript

Real-time Drilling Mud Gas Monitoring for Qualitative Evaluation of Hydrocarbon Gas Composition during Deep Sea Drilling in the Nankai Trough Kumano Basin

Sebastian B. Hammerschmidt[1§]*, Thomas Wiersberg[2]*, Verena B. Heuer[3]*, Jenny Wendt[3]*, Jörg Erzinger[2]*, Achim Kopf[1]*

[1] MARUM, University of Bremen, Leobener Str., 28359 Bremen, Germany

[2] GFZ German Research Centre for Geosciences, Telegrafenberg, 14473 Germany

*These authors contributed equally to this work

[§]Corresponding author

Email addresses:

SBH: shammerschmidt@marum.de

SUPPLEMENTARY FIGURES

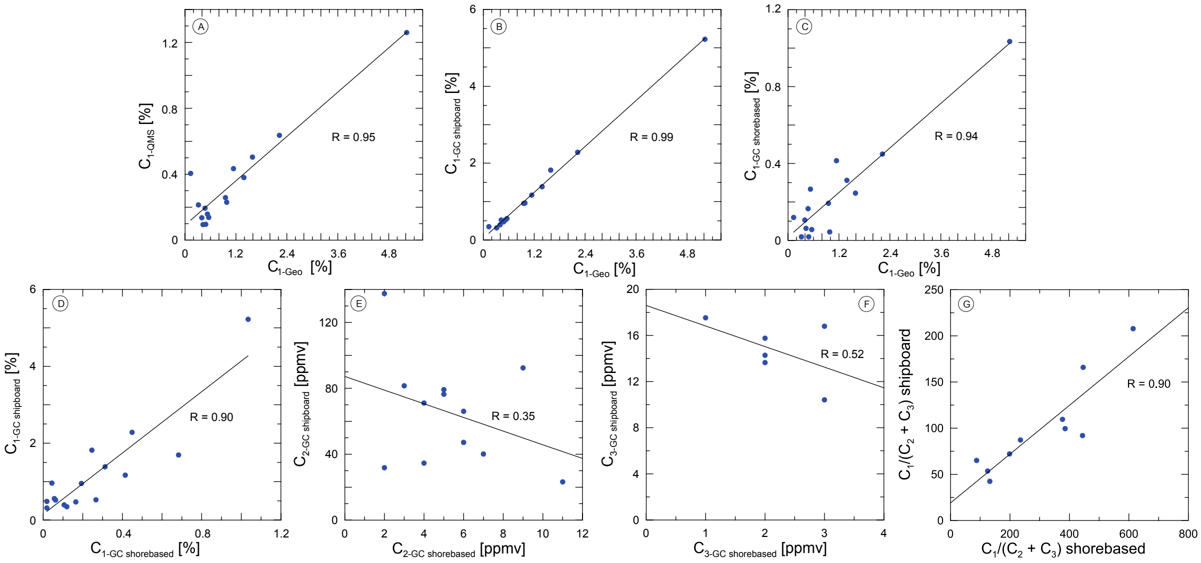

Figure S1: Correlation of SciGas (GC) and Geoscience (GEO) shipboard [1] and shorebased (GC, QMS) gas concentrations. While methane concentrations of **(A)** QMS and Geoservices **(B)** Geoservices and SciGas **(C)** GC and Geoservices and **(D)** SciGas and GC show a good correlation, **(E)** ethane and **(F)** propane concentrations show a strong scatter. The Bernard parameter **(G)** as proxy for thermal maturity, however, again shows a good correlation.

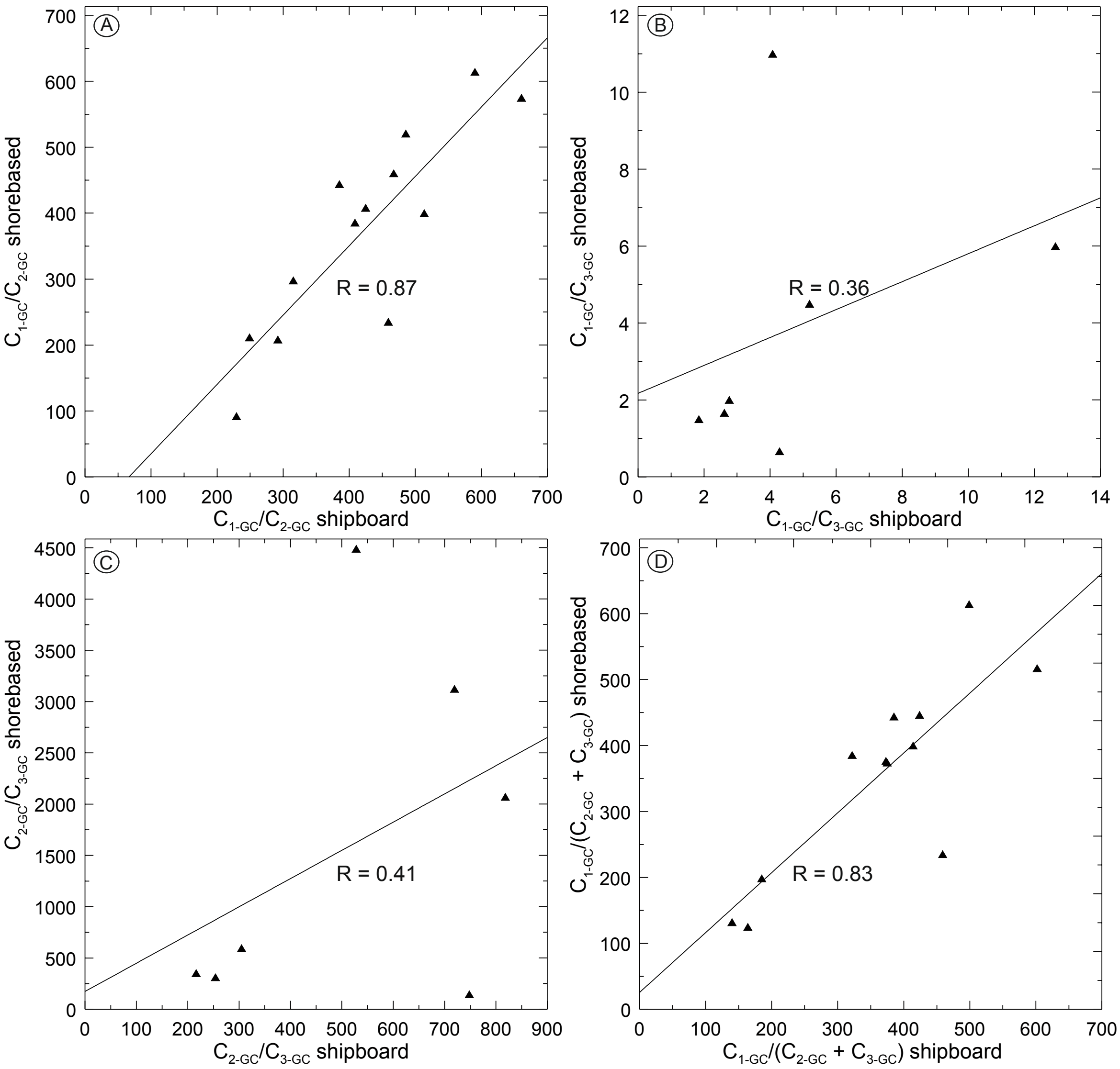

Figure S2: Correlation of SciGas shipboard and shorebased gas ratios. While the **(A)** C_1/C_2 ratios and the **(D)** Bernard parameters show a good correspondence, the data scatter of the **(B)** C_1/C_3 ratios and **(C)** C_2/C_3 ratios preclude any clear correlation.

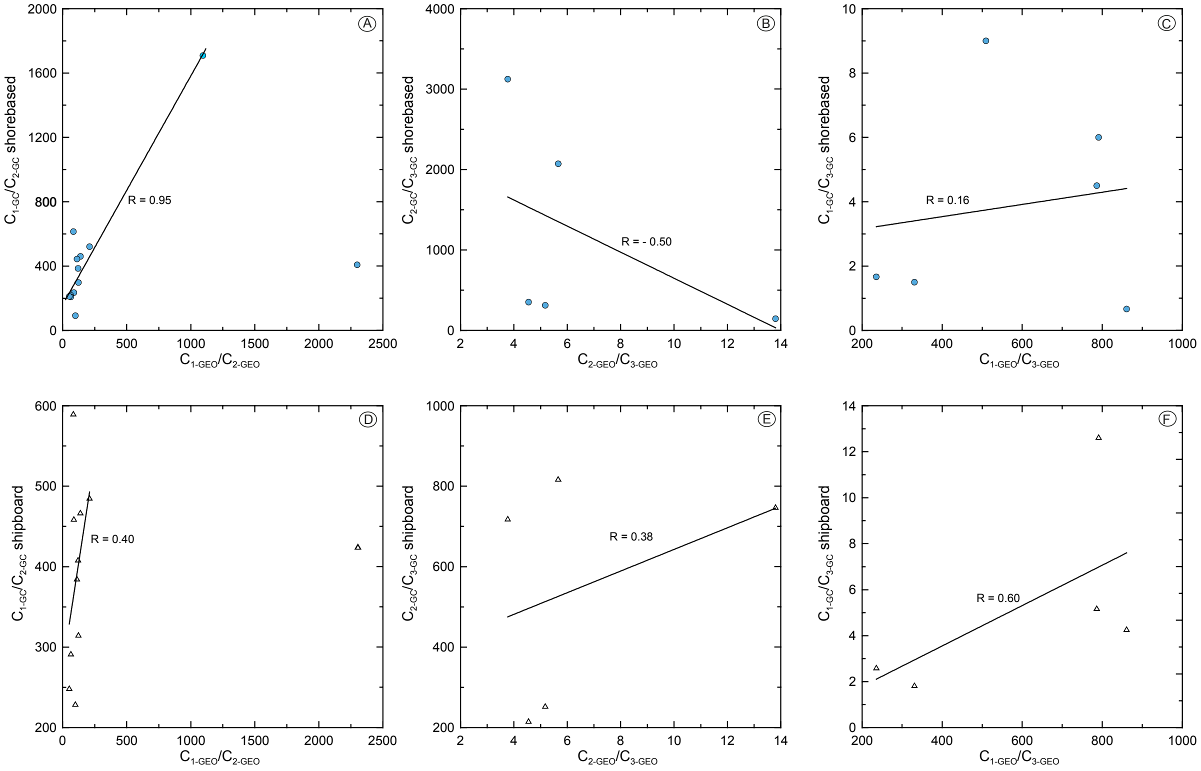

Figure S3: Correlation of SciGas shipboard and shorebased data with the dataset produced by Geoservices ("GEO") during Exp. 338 [1]. Shown are the (**A, D**) C_1/C_2, (**B, E**) C_2/C_3 and (**C, F**) C_1/C_3 ratios. "Shipboard" refers to the data obtained simultaneously with Geoservices data. "Shorebased" refers to the data gained by onshore analyses of samples taken from the SciGas system during IODP Exp. 338.

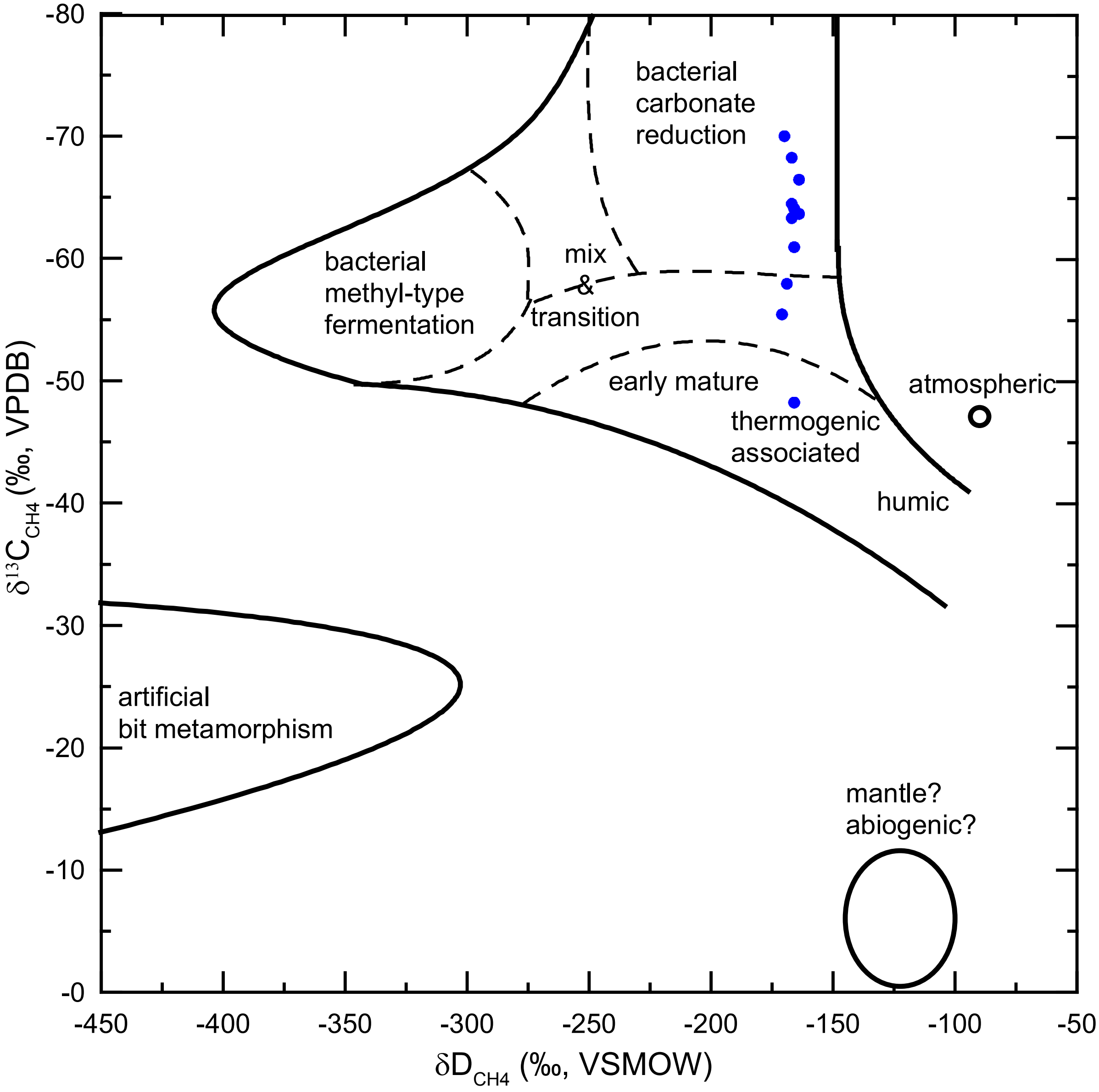

Figure S4: Shorebased δD_{CH4} values plotted against shipboard $\delta^{13}C_{CH4}$ data (diagram modified from [2]). The sampled methane is derived by bacterial carbonate reduction, and gets a more thermogenic signature with depth.

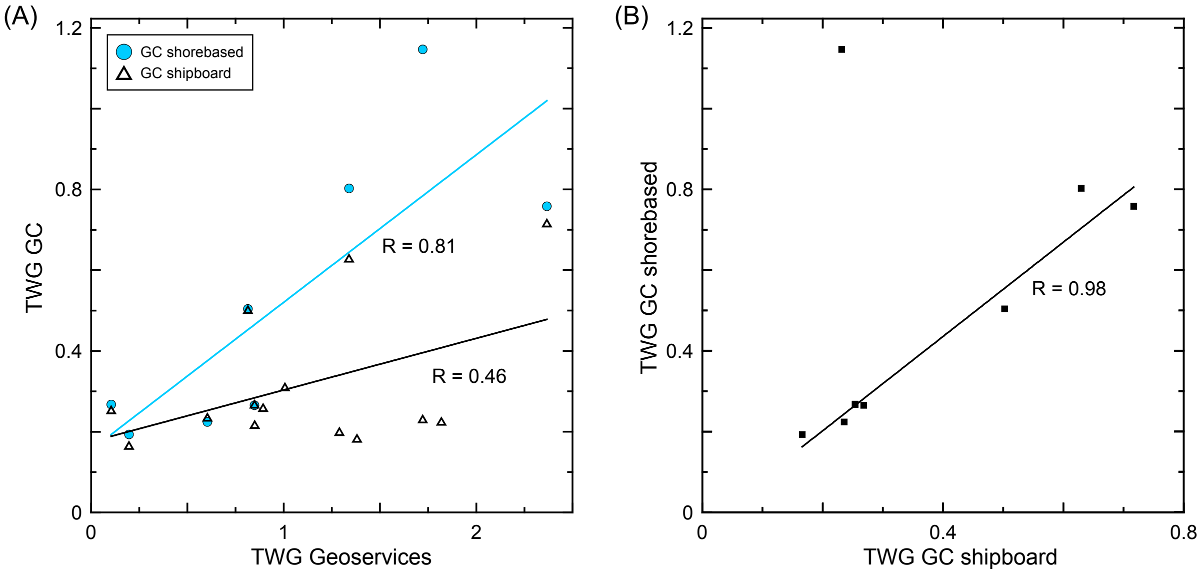

Figure S5: Correlation between the different total wet gas ratios (TWG). **(A)** The panel shows a relatively good correlation of the shorebased SciGas data with the data from Geoservices (blue circles, R = 0.81), while the two shipboard datasets reveal a larger scatter (triangles; R = 0.46). **(B)** Neglecting the outlier at ca. 1600 mbsf, the total wet gas ratios of both GC datasets correspond well with R = 0.98.

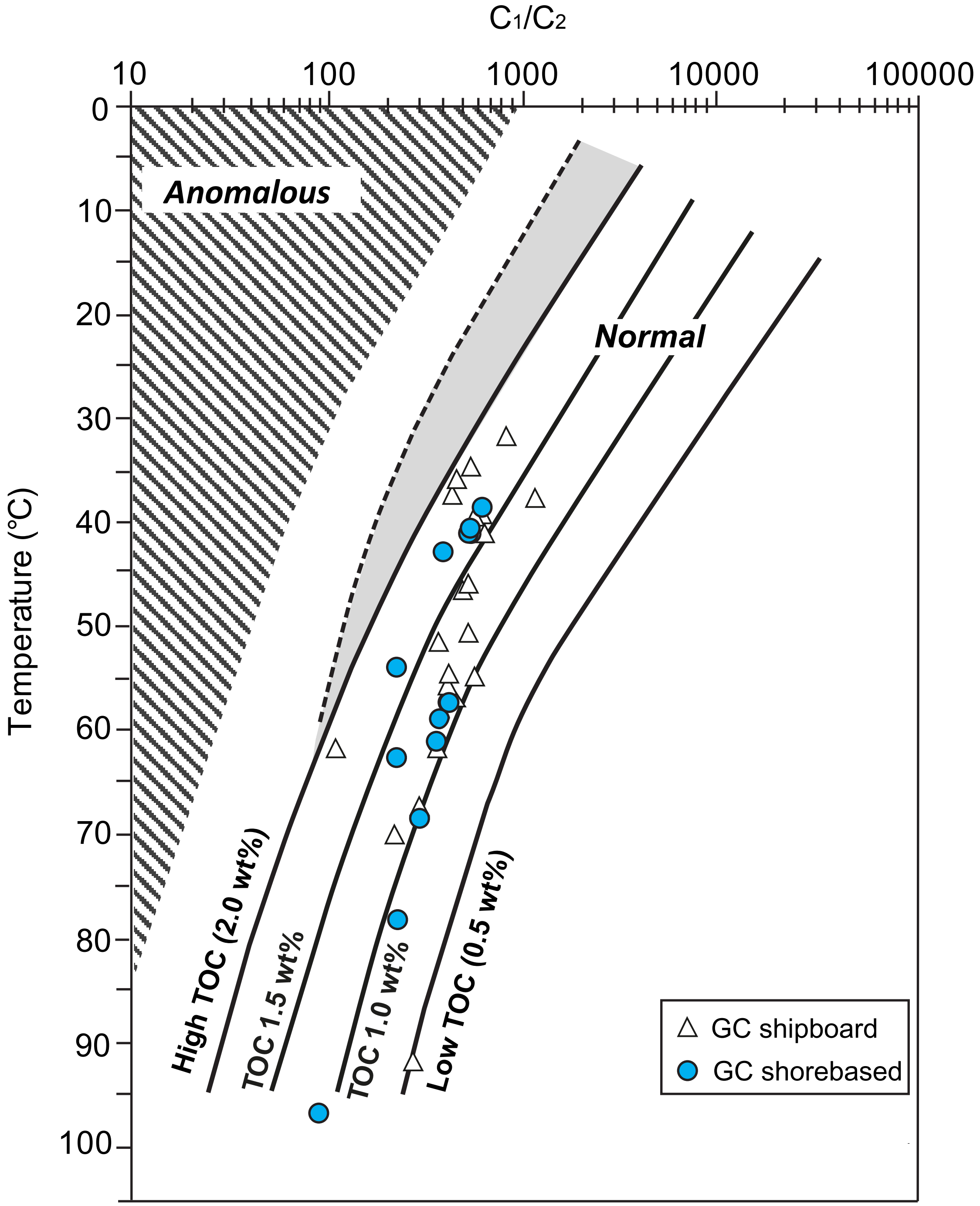

Figure S6: C_1/C_2-Temperature-TOC diagram following the empirical relationship compiled by JOIDES PPSP [3]. Shipboard TOC (= total organic carbon) data was used to associate the SciGas shipboard (white points; [1]) and the onshore GC data (blue points) to appropriate temperatures. The temperature estimations are given in **Figure 7** in the main text. See main text for comprehensive explanations.

References

1. Strasser M, Dugan B, Kanagawa K, Moore GF, Toczko S, Maeda L, the Expedition 338 Scientists: **Site C0002**. In *Proc IODP 338*. Edited by Strasser M, Dugan B, Kanagawa K, Moore GF, Toczko S, Maeda L, the Expedition 338 Scientists. Integrated Ocean Drilling Program Management International, Inc.: Tokyo; 2014a. doi:10.2204/iodp.proc.338.103.2014

2. Whiticar MJ: **Carbon and hydrogen isotope systematics of bacterial formation and oxidation of methane**. *Chem Geol* 1999, **161**:291 – 314.

3. JOIDES PPSP: **Ocean Drilling Guidelines for Pollution Prevention and Safety**. *JOIDES J* 1992, **18**.
