# Supplementary material for: Real-time drilling mud gas monitoring for qualitative evaluation of hydrocarbon gas composition during deep sea drilling in the Nankai Trough Kumano Basin
Source: Geochem Trans. 2014 Dec 16;15:15. doi: 10.1186/s12932-014-0015-8 (PMC4302130; doi:10.1186/s12932-014-0015-8)
Supplement: Additional file 3: Table S2. — Precisions for shorebased QMS and GC measurements. Please be aware that GC precision is given in relative (%) and absolute (ppmv) numbers. [file 12932_2014_15_MOESM3_ESM.pdf]

Table S2: Precisions for shorebased QMS and GC measurements. Please be aware that GC precision is given in relative (%) and absolute (ppmv) numbers

**Concentration range (ppmv)**	**GC precision**	**QMS RSD (%)**
100000 - 1000	0.50%	0.1 - 0.5
1000 - 100	0.50%	1 - 5
100 - 10	0.5 ppmv	5 - 10
< 10	0.5 ppmv	10 - 20
